# Supplementary material for: Programmed Death Ligand-1 and Tumor Burden Score Dictate Treatment Responses in Patients with Recurrent or Metastatic Head and Neck Squamous Cell Carcinoma
Source: Cancers (Basel). 2024 Apr 30;16(9):1748. doi: 10.3390/cancers16091748 (PMC11083703; doi:10.3390/cancers16091748)
Supplement: Supplementary file 1 [file cancers-16-01748-s001.zip › cancers-2972892-supplementary.pdf]

Supplementary Materials: Programmed death ligand-1 and tumor burden score dictate treatment responses in patients with recurrent or metastatic head and neck squamous cell carcinoma.

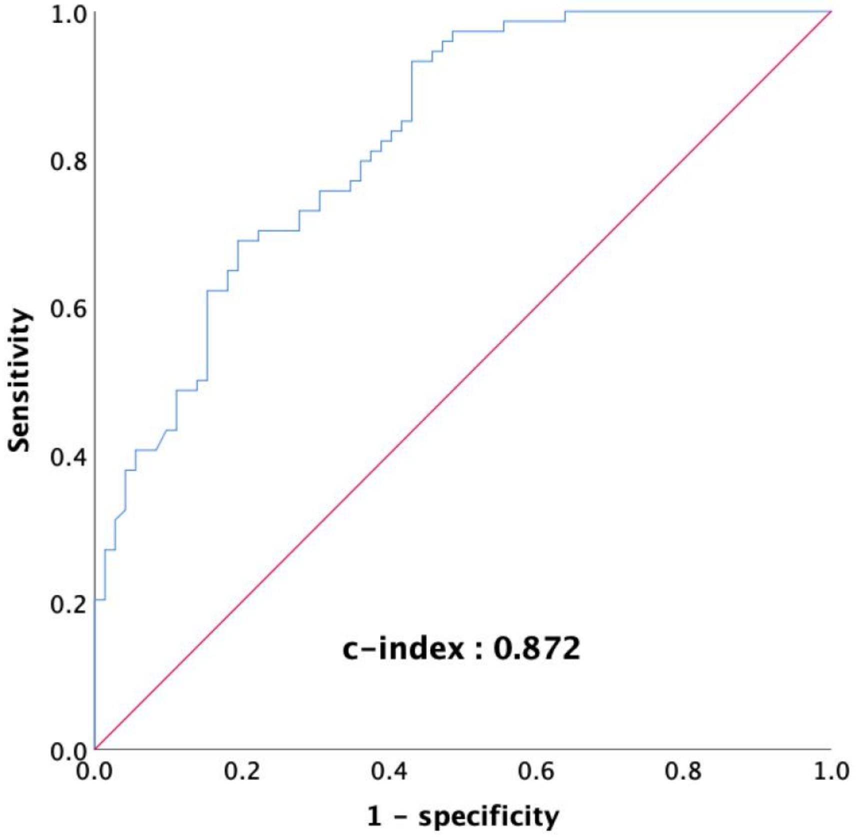

Supplementary Figure S1: Receiver operating characteristic curve.
